# Genomic Surveillance of Monkeypox Virus, Minas Gerais, Brazil, 2022

**DOI:** 10.3201/eid2906.230113

**Published:** 2023-06

**Authors:** Natália R. Guimarães, Luiz Marcelo R. Tomé, Ludmila O. Lamounier, Marcos Vinícius F. Silva, Maurício T. Lima, Alana Vitor B. da Costa, Kelly Cristina M. Luiz, Ronaldo de Jesus, Giliane de S. Trindade, Danilo B. Oliveira, Flávio G. da Fonseca, Ana Paula S.M. Fernandes, Jaqueline S. de Oliveira, Josiane B.P. Moura, Erna G. Kroon, Marta Giovanetti, Vagner Fonseca, Luiz Alcantara, Talita Emile R. Adelino, Felipe C. de Melo Iani

**Affiliations:** Ezequiel Dias Foundation, Belo Horizonte, Brazil (N.R. Guimarães, L.M.R. Tomé, L.O. Lamounier, M.V.F. Silva, M.T. Lima, A.V.B. da Costa, K.C.M. Luiz, J.B.P. Moura, T.E.R. Adelino, F.C. de Melo Iani);; Federal University of Minas Gerais, Belo Horizonte (L.M.R. Tomé, G.d.S. Trindade, F.G. da Fonseca, A.P.S.M. Fernandes, E.G. Kroon);; Brazilian Ministry of Health, Distrito Federal, Brazil (R. de Jesus);; Federal University of Vales do Jequitinhonha e Mucuri, Diamantina, Brazil (D.B. Oliveira);; State Secretary of Health of Minas Gerais, Belo Horizonte (J.S. de Oliveira);; University of Campus Bio-Medico, Rome, Italy (M. Giovanetti);; Instituto Rene Rachou, Fundação Oswaldo Cruz, Belo Horizonte (M. Giovanetti, L. Alcantara);; Pan American Health Organization, Distrito Federal, Brazil (V. Fonseca)

**Keywords:** Monkeypox virus, mpox, viruses, zoonoses, sexually transmitted diseases, genomic surveillance, Minas Gerais, Brazil

## Abstract

Phylogenetic analysis of 34 monkeypox virus genome sequences isolated from patients in Minas Gerais, Brazil, revealed initial importation events in early June 2022, then community transmission within the state. All generated genomes belonged to the B.1 lineage responsible for a global mpox outbreak. These findings can inform public health measures.

Human mpox (formerly monkeypox) is an emerging zoonotic disease caused by monkeypox virus (MPXV) (*1*,*2*). Since the 1970s, mpox outbreaks in humans have occurred sporadically, mainly in Africa (*3*). In early May 2022, mpox emerged worldwide, and case numbers increased substantially (*4*). On July 23, 2022, the World Health Organization (WHO) declared the mpox outbreak a Public Health Emergency of International Concern (*5*).

Genomic surveillance might be considered a fundamental approach to tracking circulating strains and investigating viral spread (*6*–*8*). By October 2022, Brazil had reported 12,378 mpox cases, and the state of Minas Gerais, located in southeast Brazil, reported a total of 838 cases through epidemiologic week 41 (*9*).

We selected 34 human MPXV-positive samples collected in Minas Gerais during June–September 2022 for whole-genome sequencing at the Central Laboratory of Public Health of Minas Gerais. The selected samples had cycle threshold values <30 and available epidemiologic patient data. The study was approved by the research ethics committee of the Ezequiel Dias Foundation (approval no. 62702222.6.0000.9507).

We extracted viral DNA from lesion exudate and sequenced with the Ion Torrent PGM platform (Thermo Fisher Scientific, https://www.thermofisher.com) using a set of MPXV-specific primers designed for this study by using the primalscheme platform version 1.3.2 (https://pypi.org/project/primalscheme) (Appendix Table 1). We used the MPXV reference genome (GenBank accession no. NC_063383.1) to perform genome assembly by using Burrows-Wheeler Aligner version 0.7.17 (https://github.com/lh3/bwa), SAMtools version 1.11 (https://github.com/samtools), and iVar version 1.0 (https://github.com/andersen-lab/ivar). We used Nextclade version 2.8.1 (Nextstrain, https://clades.nextstrain.org) to assess genome quality and classification. 

We used MAFFT version 7.310 (https://mafft.cbrc.jp) to align the 34 genomes obtained from this study with an additional 218 MPXV genomes collected from GISAID (https://www.gisaid.org) until October 3, 2022 (Appendix Table 2). We used BEAST version 1.10.4 (https://beast.community) to infer the Bayesian phylogeny. The Brazilian Ministry of Health Notifiable Diseases Information System provided weekly notified cases of MPXV infection in Minas Gerais. 

Epidemiologic data revealed that the highest number (n = 112) of MPXV cases in Minas Gerais were reported during epidemiologic week 31 (Appendix Figure 1). The data also highlight that the metropolitan region of Belo Horizonte had the highest concentration (n = 608) of confirmed cases during June–September (Appendix Figure 2).

Using patients’ clinical records, we found that 55.9% (19/34) were HIV-positive and 23.5% (8/34) reported active sexually transmitted infection. Among the screened samples, 33 were from male patients and 1 was from a female patient; patients were 22–46 (mean 32.5) years of age. The most frequent signs and symptoms were rash (34/34, 100%), lymphadenopathy (22/34, 64.7%), and fever (21/34, 61.8%) (Appendix Figure 3). Among mpox patients, 17 reported no travel history, 15 reported travel history to the state of São Paulo, Brazil, and 1 each reported travel to London and to Portugal.

Using the Ion Torrent PGM platform, we obtained a total number of 34 MPXV genome sequences. Genome coverage was 76.2%–97.5% (mean 87%) and had an average depth of 391× (Table). All the genomes generated in this study belonged to lineages B.1 (n = 13), B.1.1 (n = 19), B.1.2 (n = 1), and B.1.9 (n = 1), which are lineages responsible for the 2022 outbreak (*7*,*8*).

**Table:** Summary statistics of assembled genomes from genomic surveillance of monkeypox virus, Minas Gerais, Brazil, 2022*

Sample ID	Collection date	Mapped reads	Mean read depth	Coverage, %	Lineage	GISAID ID
311257928†	2022 Jun 28	241,791	208.5	90.3	B.1	EPI_ISL_13780332
311261010	2022 Jul 1	520,016	473.6	86.5	B.1.1	EPI_ISL_16650224
311261273	2022 Jul 1	478,258	447.8	90.7	B.1	EPI_ISL_16650225
311261816	2022 Jul 4	318,530	244.3	84.9	B.1	EPI_ISL_16650230
311261841	2022 Jul 4	388,417	347.0	93.5	B.1	EPI_ISL_16650229
311262116‡	2022 Jul 4	334,589	234.4	83.3	B.1	EPI_ISL_16650231
311262133	2022 Jul 4	362,965	331.9	85.3	B.1.1	EPI_ISL_16650228
311262224	2022 Jul 4	449,397	420.0	91.4	B.1	EPI_ISL_16650226
311262265	2022 Jul 4	427,206	399.6	93.6	B.1.1	EPI_ISL_16650227
311262687	2022 Jul 5	353,768	282.2	76.2	B.1.1	EPI_ISL_16650233
311262723	2022 Jul 5	342,256	266.3	81.8	B.1	EPI_ISL_16650234
311263370	2022 Jul 5	388,951	308.5	80.5	B.1.1	EPI_ISL_16650232
311265338	2022 Jul 5	344,341	299.7	82.5	B.1.1	EPI_ISL_16650238
311263885	2022 Jul 6	296,827	256.8	78.4	B.1	EPI_ISL_16650236
311263902	2022 Jul 6	394,450	332.8	79.2	B.1.1	EPI_ISL_16650235
311264859	2022 Jul 7	393,675	342.8	81.0	B.1.1	EPI_ISL_16650237
311266133	2022 Jul 8	345,305	290.7	79.1	B.1.1	EPI_ISL_16650239
311266186	2022 Jul 8	354,920	274.4	87.8	B.1	EPI_ISL_16650240
311266233	2022 Jul 8	284,916	239.2	82.9	B.1.1	EPI_ISL_16650241
311266796	2022 Jul 11	384,836	358.5	82.2	B.1.9	EPI_ISL_16650243
311267285	2022 Jul 11	351,311	325.0	79.4	B.1.1	EPI_ISL_16650242
311267311	2022 Jul 11	341,773	320.4	81.0	B.1.1	EPI_ISL_16650244
311267938	2022 Jul 12	330,526	300.1	77.1	B.1.1	EPI_ISL_16650245
311271087§	2022 Jul 15	581,432	594.2	97.5	B.1.1	EPI_ISL_16650246
311283035	2022 Aug 5	590,550	584.0	96.3	B.1	EPI_ISL_16650248
311287351	2022 Aug 12	565,565	557.1	96.5	B.1.1	EPI_ISL_16650247
311288391	2022 Aug 15	533,148	528.6	97.1	B.1.1	EPI_ISL_16650249
311291580	2022 Aug 22	520,820	554.3	91.7	B.1	EPI_ISL_16650262
311294876	2022 Aug 26	567,700	528.8	97.0	B.1	EPI_ISL_16650251
311297067	2022 Aug 31	539,498	454.0	94.7	B.1.2	EPI_ISL_16650253
311300630	2022 Sep 8	532,582	549.0	88.9	B.1.1	EPI_ISL_16650258
311300699	2022 Sep 8	476,181	514.3	82.5	B.1	EPI_ISL_16650255
311303564	2022 Sep 13	533,191	563.8	95.9	B.1.1	EPI_ISL_16650260
311309205	2022 Sep 26	546,501	564.2	92.9	B.1.1	EPI_ISL_16650265

*Genome assembly performed by using Burrows-Wheeler Aligner version 0.7.17 (https://github.com/lh3/bwa) and iVar version 1.0 (https://github.com/andersen-lab/ivar) pipeline and lineage ID was assigned to each genome by using Nextclade version 2.8.1 (Nextstrain, https://clades.nextstrain.org). GISAID, https://www.gisaid.org. ID, identification. †First confirmed mpox case in Minas Gerais; patient had travel history to London, UK. ‡Patient had travel history to Portugal and São Paulo, Brazil. §First mpox death reported in Minas Gerais.

Our phylogenetic reconstruction revealed that all genomes from the 2022 mpox outbreak grouped together (Figure). Most of the genomes we obtained from Minas Gerais grouped with MPXV genomes isolated from other regions of Brazil (Figure). Our phylogenetic reconstruction revealed that the first mpox case reported in Minas Gerais, isolated from a patient with a travel history to London, UK (GISAID accession no. EPI_ISL_13780332), grouped with a genome sequence from the United Kingdom (GISAID accession no. EPI_ISL_14439774).

**Figure:**
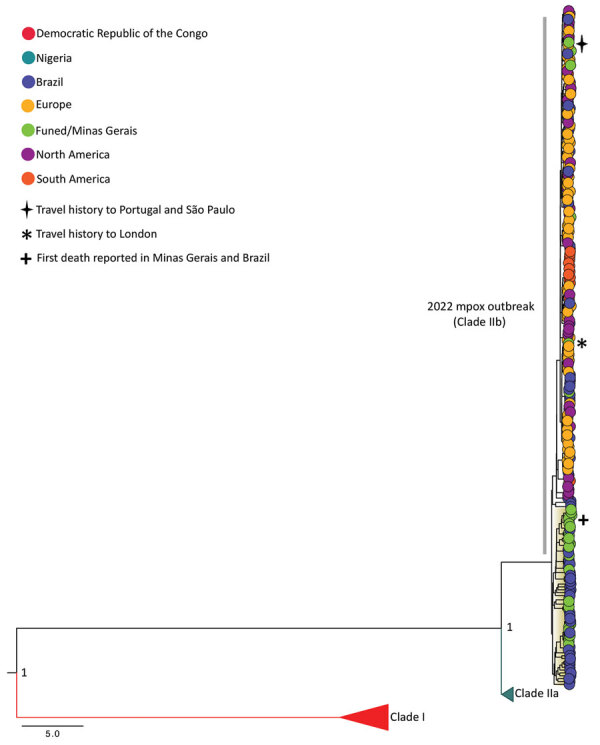
Bayesian phylogenetic tree of 34 genome sequences generated during genomic surveillance of monkeypox virus, Minas Gerais, Brazil, 2022. The tree also includes 218 reference strains available at GISAID (https://www.gisaid.org), accessed October 3, 2022. Colors represent different sampling locations. Posterior probability support is shown at key nodes for clade I, IIa, and IIb. Scale bar indicates nucleotide substitutions per site.

We also sequenced a sample from the first confirmed mpox death in Brazil, which was reported in late July 2022. That sample was collected from a patient who resided in Minas Gerais and was in treatment for diffuse large B-cell lymphoma and HIV (*10*). The genome from that patient’s sample belonged to the B.1.1 lineage, and in our phylogenetic reconstruction, it clustered with genome sequences isolated from Minas Gerais and from other states in Brazil.

Overall, our data revealed that an mpox case detected in Minas Gerais in early 2022 was related to a likely importation event, probably associated with a traveler returning from the United Kingdom, and then sustained MPXV community transmission. The first confirmed death reported in Minas Gerais was associated with a local MPXV infection described in a patient who reported several underlying conditions. These results contribute to genomic MPXV surveillance in Minas Gerais and increase the number of genome sequences from this virus available in GISAID. These findings and the available data can help future studies aiming to improve diagnostic protocols and vaccine development.

Appendix Additional information on genomic surveillance of monkeypox virus, Minas Gerais, Brazil.
