# Supplementary material for: Genomic Surveillance of Monkeypox Virus, Minas Gerais, Brazil, 2022
Source: Emerg Infect Dis. 2023 Jun;29(6):1270–3. doi: 10.3201/eid2906.230113 (PMC10202877; doi:10.3201/eid2906.230113)
Supplement: Appendix — Additional information on genomic surveillance of monkeypox virus, Minas Gerais, Brazil. [file 23-0113-Techapp-s1.pdf]

Article DOI: https://doi.org/10.3201/eid2906.230113

EID cannot ensure accessibility for supplementary materials supplied by authors. Readers who have difficulty accessing supplementary content should contact the authors for assistance.

Genomic Surveillance of Monkeypox Virus, Minas Gerais, Brazil

Appendix

Appendix Table 1. Set of monkeypox virus–specific primers designed to generate complete genomes (https://github.com/genomicsurveillance/MPXV_Primer_Schemes).

Primer name	Pool no.	Sequence
monkeypox_1_LEFT	1	AAAAATGTGTGACCCACGACCG
monkeypox_1_RIGHT	1	AGCAACTTCAGGATTCTTTACAGGA
monkeypox_2_LEFT	2	GCCCACTAGAGTTACCGTCTCT
monkeypox_2_RIGHT	2	GCTCTTTTATCTCGAGGAGCCG
monkeypox_3_LEFT	1	TGAGAGAGCGACTTCCATATCCT
monkeypox_3_RIGHT	1	TGTGGTATCCCAGATGAAAATGATGT
monkeypox_4_LEFT	2	TACTGCGTTGACATTTGCTCCA
monkeypox_4_RIGHT	2	AACGTTACACACAAGGGAGTCG
monkeypox_5_LEFT	1	TGTAACTTCGTCGTACAGCGTT
monkeypox_5_RIGHT	1	GTGTCCTCTCCTCTTATAACATCGT
monkeypox_6_LEFT	2	GTCTGTAGACCTTTATCGTCGTACA
monkeypox_6_RIGHT	2	ACCTACATTTCTAATCTTCCGTATACCA
monkeypox_7_LEFT	1	CATACGTTCCACACATCCGGAA
monkeypox_7_RIGHT	1	ACACGCATACCTTTTCAATGAGC
monkeypox_8_LEFT	2	GTAGCTTCTAGTAGAGCCATCGC
monkeypox_8_RIGHT	2	TTGACAAATGCGATATTAAACGGGA
monkeypox_9_LEFT	1	TGTATCATGGGCAGATAATAGTTTATCCA
monkeypox_9_RIGHT	1	TGGGTATACAACACGAATTCGACA
monkeypox_10_LEFT	2	CTTTGACCTCGTCGATTTCCGA
monkeypox_10_RIGHT	2	GCTTTGTCGATATATGCATGGATCA
monkeypox_11_LEFT	1	GTAAAGACAGTACACGGGTCCG
monkeypox_11_RIGHT	1	AGACATCTCAAAAATCTTACAGATGATGA
monkeypox_12_LEFT	2	ATGGCAAATCTAACTGCGGGTT
monkeypox_12_RIGHT	2	AATCTTTTACTTCCATCAACGGATGT
monkeypox_13_LEFT	1	GGATCCCACTTCAACAGCCATC
monkeypox_13_RIGHT	1	TCCCTTCTCTTCATTCCGGACA
monkeypox_14_LEFT	2	AGTCTGAGTTCCTCGTTTTTGCT
monkeypox_14_RIGHT	2	GTCGCTATTGTCTGCATCAGGT
monkeypox_15_LEFT	1	ACACACAGTACTATCGACGAAACT
monkeypox_15_RIGHT	1	TCCGGATAATACAATCGCAGTGA
monkeypox_16_LEFT	2	GCCGAAATTCGTACCCACTTCA
monkeypox_16_RIGHT	2	AGGGTTTGGGTGTAAGATTGGC
monkeypox_17_LEFT	1	TATTCACGCGTGCTATGGATGG
monkeypox_17_RIGHT	1	TGTCATTAAGTGGTATATGAGGCGA
monkeypox_18_LEFT	2	ACTTGTATGATTTGCGTGGTTGT
monkeypox_18_RIGHT	2	TGATAATGCATCTTCCGTGCCC
monkeypox_19_LEFT	1	TTGTATATCGCTATCGCCGCAA
monkeypox_19_RIGHT	1	CGATTGCCGTATTACGATCCGT
monkeypox_20_LEFT	2	GATCCCTCCATTATCGCGATCG
monkeypox_20_RIGHT	2	TGTTGCCAATGATTCATCCCCT
monkeypox_21_LEFT	1	CGTTTATCTTTTTGTTGGAAAAAGGATGA
monkeypox_21_RIGHT	1	TCCGAGAATACTGGAATGGGGA
monkeypox_22_LEFT	2	GACGTCCTGCAGGGTATTGTTT
monkeypox_22_RIGHT	2	GCCAGTTAGCACTGCGTATCAT
monkeypox_23_LEFT	1	GTGTAGTGGGAACTATGGCCAA
monkeypox_23_RIGHT	1	ACAATTTTGGAAGTGTCGATGCA
monkeypox_24_LEFT	2	ATCCAATTGAGAAGCGCATTTAGT
monkeypox_24_RIGHT	2	ACGTCGCTGTAATAGACAAGGC

Primer name	Pool no.	Sequence
monkeypox_25_LEFT	1	TGAAGAATATCTATATCACCGTACCGT
monkeypox_25_RIGHT	1	AGACATCACAAAGACAGAGTTGGA
monkeypox_26_LEFT	2	AGAGATGTAACGGGAACAGGGT
monkeypox_26_RIGHT	2	TTAGACACGCGTGTAGAGATTGT
monkeypox_27_LEFT	1	CATCATCCTCTGGTGGTTCGTC
monkeypox_27_RIGHT	1	ATGGGTTCCCCTCCGTGAAATA
monkeypox_28_LEFT	2	CGGTTTTACGACACGCATTTGA
monkeypox_28_RIGHT	2	AACAGACGCGTTACATTGCCTA
monkeypox_29_LEFT	1	AGTTCTCCAGAAACGCAACAAATG
monkeypox_29_RIGHT	1	AGCGTGTATGGAGATACCGACT
monkeypox_30_LEFT	2	TTTGAATTCGACGATGCCGAGT
monkeypox_30_RIGHT	2	CGATCCACGTAACTGTTTCGCT
monkeypox_31_LEFT	1	ACATCCTCTATCGACGGCTTCT
monkeypox_31_RIGHT	1	TGCTGTCTGTAATTGCTCCAGA
monkeypox_32_LEFT	2	CAATGGACCCCAACATCGTTGA
monkeypox_32_RIGHT	2	AGCGTTTGTCTTCTGTAGTTGGT
monkeypox_33_LEFT	1	CCAGTGCGTTTCTACAAAAAGGA
monkeypox_33_RIGHT	1	TCCACTTTTATTATGTTGGAAGGAGAGA
monkeypox_34_LEFT	2	TTCCGCCGAAAGACTATGCAAA
monkeypox_34_RIGHT	2	GGAAACAAACGGCCTGGAGTTA
monkeypox_35_LEFT	1	CGAACTCGTCTCCCCAAACATT
monkeypox_35_RIGHT	1	TTTCGTTTTTCCCATTCGATACAGA
monkeypox_36_LEFT	2	TGACGCTAGTGGAATTGAAGATTCT
monkeypox_36_RIGHT	2	TGTAAGAGAATACATTAACGCAGTTTGG
monkeypox_37_LEFT	1	ACTTTGCCAGCGGTTGTAGATT
monkeypox_37_RIGHT	1	TCCCGTCCTCTCAAAGTTATCCA
monkeypox_38_LEFT	2	TCTAACACTCCCCGAAGATTTGT
monkeypox_38_RIGHT	2	GGAGTCAGACGCAATCAGAACA
monkeypox_39_LEFT	1	GCCTCCGCTAAGAAACGTTAGT
monkeypox_39_RIGHT	1	AAAAAGGGTTACATGTCCTCCCT
monkeypox_40_LEFT	2	ACTGCTGGAAAATAAATCACTGACG
monkeypox_40_RIGHT	2	AACAACGGAGGAGTAGTTATGCA
monkeypox_41_LEFT	1	TGCCGGTATTGATACATCGTCT
monkeypox_41_RIGHT	1	TGACTCTCTTTTTGAATCCTCGTAGT
monkeypox_42_LEFT	2	TTAGTCAGCATCAGCATTTGGC
monkeypox_42_RIGHT	2	TGGTGGATGTGAACAGCATACG
monkeypox_43_LEFT	1	GCCCCAGGATCTCCAACAAATT
monkeypox_43_RIGHT	1	GGCATAACCTCATTGTTAGGATCCA
monkeypox_44_LEFT	2	ACAAATGGTTATTGCTGGTGCA
monkeypox_44_RIGHT	2	AGTCGATGTAACACTTTCTACACACA
monkeypox_45_LEFT	1	TGGGACATTTCAACGTAGACCG
monkeypox_45_RIGHT	1	TCTGTGATTCCAAACGAGTAGCT
monkeypox_46_LEFT	2	GGTAACTGGAGTAGAGATAGCCGA
monkeypox_46_RIGHT	2	ACGCTGCTACGATTTCATCTTGA
monkeypox_47_LEFT	1	CCCGGAATTGCCAACTCTCAAA
monkeypox_47_RIGHT	1	CCTCGTCCTCAATGAATGTCTCC
monkeypox_48_LEFT	2	TCCAGACATCACTCTTCGAAAAGA
monkeypox_48_RIGHT	2	ACAGGAACATTTGTTACCGCATTTA
monkeypox_49_LEFT	1	TATCTGCCGCAAGCTATTAGGC
monkeypox_49_RIGHT	1	CACTCTTAAATACGCCATCTCCGT
monkeypox_50_LEFT	2	TGGGTTCTAATGCCGATCTCTG
monkeypox_50_RIGHT	2	CGTAATTGACCACGCCATTACG
monkeypox_51_LEFT	1	AGTCCATTTAAAGTTACAAACAACTAGGA
monkeypox_51_RIGHT	1	CCAGATATCGGAGTGAGGGTCA
monkeypox_52_LEFT	2	ATGACAGCTTTCTTCGGAGAGC
monkeypox_52_RIGHT	2	TGCTTTGATCTCTTAAATGTTCGACT
monkeypox_53_LEFT	1	AGTGGGTATTAAGTCCGTTGTTGT
monkeypox_53_RIGHT	1	TGACCATTTGTCTGGCTACTGA
monkeypox_54_LEFT	2	TGCTGGAAGACAAGTTAGATGGT
monkeypox_54_RIGHT	2	TGACCAGTGGTACCGTTGTAAA
monkeypox_55_LEFT	1	TGTAATGCAACCACCTCACGAT
monkeypox_55_RIGHT	1	AAGCTACCAAGGCGAACAACAA
monkeypox_56_LEFT	2	AGGATATTTCATCTCCGTAAATATATGCC
monkeypox_56_RIGHT	2	AACGTCAACTCATGTGGTTCCA
monkeypox_57_LEFT	1	CCGTTTAGACTTTCCTACGAACCAT
monkeypox_57_RIGHT	1	ACTGTTATATGTTCATCACTTTCCTCTT
monkeypox_58_LEFT	2	AAGAACTTCATTCGCATTCCAGG
monkeypox_58_RIGHT	2	GGGATGCTATTGCGGCTGATA
monkeypox_59_LEFT	1	TCTTGGTTAAACTCAGCCACCG

Primer name	Pool no.	Sequence
monkeypox_59_RIGHT	1	TGCCGACCGACATGTTAAAACT
monkeypox_60_LEFT	2	GAACAGTTTATTGCGTGCGCTG
monkeypox_60_RIGHT	2	CTTTGATCGGTGGGGATGACTT
monkeypox_61_LEFT	1	GCACGTAACCGAATCTTCCCAT
monkeypox_61_RIGHT	1	TGGACCTTGGATGTACGATCCT
monkeypox_62_LEFT	2	TGAGGCAGCAAATACTGAACCA
monkeypox_62_RIGHT	2	TTCAGGCCGAAACAAACGATCT
monkeypox_63_LEFT	1	GCTATTGACCACGGCTTCCATT
monkeypox_63_RIGHT	1	ACACCATTTCTTCTGAAAGTTTGGT
monkeypox_64_LEFT	2	AGGCATCCAACATCGACATTCG
monkeypox_64_RIGHT	2	ACATTTTTAGTTATTGCCATTAATGCCATG
monkeypox_65_LEFT	1	TCCCCAAAGGATCAATGCATGT
monkeypox_65_RIGHT	1	GGAAGTTCTGATTCCACAGCCA
monkeypox_66_LEFT	2	TCATCTGGCATTTTTAATATCCGTCA
monkeypox_66_RIGHT	2	GTTACCCAGAGTAGTCGGAGGT
monkeypox_67_LEFT	1	ATCAGCCATGAGCCTGAGTAGT
monkeypox_67_RIGHT	1	ACCACTTCATCCTCTAGCATCTG
monkeypox_68_LEFT	2	CGCCACCTCCTACTTTTCGTAG
monkeypox_68_RIGHT	2	GCCGTTGCTGCTGTTAGTTATAAC
monkeypox_69_LEFT	1	GCGATTAGCACGCCAGAAAAAT
monkeypox_69_RIGHT	1	GGTTTGATTTTGTTCGTCTTGGC
monkeypox_70_LEFT	2	TAGACATTCCCACAGTGTTGCC
monkeypox_70_RIGHT	2	TGTATGACGTGTTCTGCCTGTC
monkeypox_71_LEFT	1	TTCCGTACCAACATCCTCCAGA
monkeypox_71_RIGHT	1	CTATGCGCCTTTCCCAATCAGA
monkeypox_72_LEFT	2	TGGAAACTTCAACCAGTTCGTCT
monkeypox_72_RIGHT	2	ACGTGGCTAATGCGTCATAACT
monkeypox_73_LEFT	1	TTTGGTGCCTGATCCTAAAGCC
monkeypox_73_RIGHT	1	CCAAGAATAGCTCTGGGTCCAG
monkeypox_74_LEFT	2	AGACGACATGACATTCTCTGACA
monkeypox_74_RIGHT	2	AGAAACGTGATCGGTATCTTTCGT
monkeypox_75_LEFT	1	CAGAGAGTTGTGGATGTTCGGG
monkeypox_75_RIGHT	1	GGAACGAGAAATGCGGTCAGAA
monkeypox_76_LEFT	2	TACGAAGTCTGGACAGTTCCCA
monkeypox_76_RIGHT	2	TGTAGAAGAGCCTGATCCTGTCA
monkeypox_77_LEFT	1	ACAGTAGGTAATGGCCATGGGA
monkeypox_77_RIGHT	1	CAGGCAGAAGTTGGACCCAATA
monkeypox_78_LEFT	2	ATTGCAAGATCGTCATCTCCGG
monkeypox_78_RIGHT	2	TGCTCCACTACTTGTCACTCCA
monkeypox_79_LEFT	1	GGGAATGTTATTTGTATTTTATAAGCCAAAGC
monkeypox_79_RIGHT	1	GCAAGCACTAGGCATCAGTTCT
monkeypox_80_LEFT	2	AGGATACATGGGGATCTGATGGT
monkeypox_80_RIGHT	2	TGCCGAATACAGGGAATATCTCC
monkeypox_81_LEFT	1	GCAAGTCAAATCCGTTCATTGCA
monkeypox_81_RIGHT	1	TGTTGATATACCTCTGTACTTTTGTGGT
monkeypox_82_LEFT	2	TCCGATAACGAATGAAGTCTAGCA
monkeypox_82_RIGHT	2	GGGGAATAACTTCACCCGGATT
monkeypox_83_LEFT	1	TGCTAATGTCAAGTTTATTCGAATAGATGT
monkeypox_83_RIGHT	1	GTATGGCCGAGCAACTGGTTAT
monkeypox_84_LEFT	2	CTATGCACAGCGTCATCATCGA
monkeypox_84_RIGHT	2	TGAGACTCCTTCATTGTGTTACAGA
monkeypox_85_LEFT	1	ACATGTTCTGCCGTAACCGATAG
monkeypox_85_RIGHT	1	AGCATCTACCATATTTTCTAGGTTATCCT
monkeypox_86_LEFT	2	CATGTGTAGATCAAACTTGGATAATCCA
monkeypox_86_RIGHT	2	CCATTCGCTGTATGTGACTTGG
monkeypox_87_LEFT	1	CGACGATGAATCCGGTAAATGGA
monkeypox_87_RIGHT	1	TCTATGACATTTCCACATACACTAGTCT
monkeypox_88_LEFT	2	CTATGATAGGATTGTGTGCGTGC
monkeypox_88_RIGHT	2	TAACTCGACGGTACATGGTTGG
monkeypox_89_LEFT	1	ACTGTATCGATTTCTACATATACGCTGA
monkeypox_89_RIGHT	1	GTTCCGGAATCTCGTCTGTTGT
monkeypox_90_LEFT	2	AGAAACTATTTCTGAGAAACCAGAGGA
monkeypox_90_RIGHT	2	GGCAAGATACCTCCCAACCATC
monkeypox_91_LEFT	1	GTCTAACGGCGAACATGTTCCA
monkeypox_91_RIGHT	1	ATGTGACACCCATTCATCTGGA
monkeypox_92_LEFT	2	ACTTCCTGTTGTGCATTTCTGC
monkeypox_92_RIGHT	2	TCACGAAAGAAGGATGTCTACCG
monkeypox_93_LEFT	1	ACTGCGTGTATGACCGATTATGA
monkeypox_93_RIGHT	1	TCTATCGTCGTTAAACGCGCTT

Primer name	Pool no.	Sequence
monkeypox_94_LEFT	2	ACGTGTTAAACAATGGGTGATGG
monkeypox_94_RIGHT	2	TCCCCATGTTTCCTCTTCAGTC
monkeypox_95_LEFT	1	TTCTCGGTAGCACATCGAATGA
monkeypox_95_RIGHT	1	TCCGATAATCCATTTCTTACCATCGT
monkeypox_96_LEFT	2	AACATTAACAAGAATCATCCCAGTGAA
monkeypox_96_RIGHT	2	GTTGATGCACAGTCTGCCACTA
monkeypox_97_LEFT	1	AGCTACGTTTATCGATGTGCACA
monkeypox_97_RIGHT	1	TGATGCTAGGAATAGTAAACGCCA
monkeypox_98_LEFT	2	GGACACCGACGTCTTTTGGATA
monkeypox_98_RIGHT	2	AAATACAAAGTCGCCTCGGTGA
monkeypox_99_LEFT	1	CCGTCACAAGACCACAGGTTTA
monkeypox_99_RIGHT	1	GCTCCTCGATCTAATAAGTACTCAGC
monkeypox_100_LEFT	2	AGAGATCGAGTCATGCACGATT
monkeypox_100_RIGHT	2	ACTCGACAAATTGGTATCGTGTACA
monkeypox_101_LEFT	1	TGACGGATGGGAATTGGTAACG
monkeypox_101_RIGHT	1	GCACGACCTATAGTAAACCGTGA
monkeypox_102_LEFT	2	TTACATTCCCGTTATTCCGCCC
monkeypox_102_RIGHT	2	TGTCGTCAGATCGTCTACGTCT
monkeypox_103_LEFT	1	TGAAGTCATTAACAACATAACTCTGACA
monkeypox_103_RIGHT	1	CTATGGGGCAAGCCACCTTTAG
monkeypox_104_LEFT	2	AAAATGTTTGACACCCGGTGAC
monkeypox_104_RIGHT	2	TCGAATGTGTAAGGAGGTTGCT
monkeypox_105_LEFT	1	TGCTGATAAACCACGTGCTTGT
monkeypox_105_RIGHT	1	TGTTCACATTTCATAGACACAACGA
monkeypox_106_LEFT	2	GACATGAATGGCGTCGCTTATC
monkeypox_106_RIGHT	2	AGGGTGAGATAGTCGTTCTCGT
monkeypox_107_LEFT	1	GCGAGTTGAAGGAGTTCTCGAA
monkeypox_107_RIGHT	1	AAGAGATCTTGCACAGAATTACAATTCT
monkeypox_108_LEFT	2	CCTTGAACCAATTTGTGATCCTGT
monkeypox_108_RIGHT	2	AGATGAATCGCTTCTCAGCTCG
monkeypox_109_LEFT	1	AACGGACCCCATTGATGACTTT
monkeypox_109_RIGHT	1	TATTGCTGGTTACGACGGGTTC
monkeypox_110_LEFT	2	AGGAGCATCAGGGTGTAGAACA
monkeypox_110_RIGHT	2	TGATGCAATTGTCTGACAACCTAGA
monkeypox_111_LEFT	1	AGCAACTTCAGGATTCTTTACAGGA
monkeypox_111_RIGHT	1	AAAAATGTGTGACCCACGACCG

Appendix Table 2. Genomic sequences obtained from Global Initiative on Sharing Avian Influenza Data (GISAID - EpiPox™ database) used in a study of genomic surveillance of monkeypox virus, Minas Gerais, Brazil.

Sample no.	Virus name	Accession no.	Collection date
1	hMpxV/Austria/MUW_1527495/2022	EPI_ISL_13573943	2022-06-21
2	hMpxV/Austria/MUW_1525179/2022	EPI_ISL_13842269	2022-06-10
3	hMpxV/Austria/MUW-1533374/2022	EPI_ISL_14166709	2022-07-11
4	hMpxV/Austria/MUW-1532243/2022	EPI_ISL_14167574	2022-07-06
5	hMpxV/Austria/MUW-1533948/2022	EPI_ISL_14934116	2022-07-13
6	hMpxV/Austria/MUW-1538395/2022	EPI_ISL_14934140	2022-08-01
7	hMpxV/Austria/MUW-1539124/2022	EPI_ISL_14934382	2022-08-02
8	hMpxV/Austria/MUW-1540386/2022	EPI_ISL_14934478	2022-08-08
9	hMpxV/Belgium/UZ_Rega_1/2022	EPI_ISL_13052282	2022-05-19
10	hMpxV/Belgium/UZ_Rega_2/2022	EPI_ISL_13052283	2022-05-22
11	hMpxV/Belgium/UZ_REGA-3/2022	EPI_ISL_13537923	2022-05-22
12	hMpxV/Belgium/UZ_REGA-4/2022	EPI_ISL_13537924	2022-05-22
13	hMpxV/Belgium/UZ_REGA-5/2022	EPI_ISL_13537925	2022-05-22
14	hMpxV/Belgium/UZ_REGA-6/2022	EPI_ISL_13537926	2022-05-22
15	hMpxV/Brazil/SP-IAL-01/2022	EPI_ISL_13191438	2022-06-07
16	hMpxV/Brazil/RS-IAL-02/2022	EPI_ISL_13234112	2022-05-31
17	hMpxV/Brazil/SP-IAL-03/2022	EPI_ISL_13314740	2022-06-11
18	hMpxV/Brazil/RS-IAL-05/2022	EPI_ISL_13343697	2022-06-07
19	hMpxV/Brazil/SP-IAL-06/2022	EPI_ISL_13343718	2022-06-15
20	hMpxV/Brazil/SP-IAL-07/2022	EPI_ISL_13436658	2022-06-14
21	hMpxV/Brazil/SP-IAL-08/2022	EPI_ISL_13436792	2022-06-13
22	hMpxV/Brazil/SP-IAL-09/2022	EPI_ISL_13437056	2022-06-20
23	hMpxV/Brazil/SP-IAL-10/2022	EPI_ISL_13459346	2022-06-21

Sample no.	Virus name	Accession no.	Collection date
24	hMpxV/Brazil/SP-IAL-11/2022	EPI_ISL_13459347	2022-06-22
25	hMpxV/Brazil/SP-IAL-12/2022	EPI_ISL_13459482	2022-06-22
26	hMpxV/Brazil/SP-IAL-13/2022	EPI_ISL_13459483	2022-06-23
27	hMpxV/Brazil/RJ-FIOCRUZ-14992/2022	EPI_ISL_13484458	2022-06-16
28	hMpxV/Brazil/SP-IAL-14/2022	EPI_ISL_13508393	2022-06-24
29	hMpxV/Brazil/SP-IAL-15/2022	EPI_ISL_13508471	2022-06-24
30	hMpxV/Brazil/SP-IAL-16/2022	EPI_ISL_13705358	2022-07-04
31	hMpxV/Brazil/SP-IAL-17/2022	EPI_ISL_13705407	2022-07-04
32	hMpxV/Brazil/SP-IAL-18/2022	EPI_ISL_13732932	2022-07-06
33	hMpxV/Brazil/SP-IAL-21/2022	EPI_ISL_14021725	2022-07-13
34	hMpxV/Brazil/SP-IAL-25/2022	EPI_ISL_14070855	2022-07-19
35	hMpxV/Brazil/PR-IAL-26/2022	EPI_ISL_14414948	2022-08-01
36	hMpxV/Brazil/SP-IAL-27/2022	EPI_ISL_14415810	2022-08-03
37	hMpxV/Brazil/RS-CEVS-CDCT60/2022	EPI_ISL_14465517	2022-08-04
38	hMpxV/Brazil/AM-FIOCRUZ-ILMD2204652/2022	EPI_ISL_14467428	2022-07-28
39	hMpxV/Brazil/AM-FIOCRUZ-ILMD2204653/2022	EPI_ISL_14467429	2022-08-04
40	hMpxV/Brazil/SP-IAL-30/2022	EPI_ISL_14571435	2022-08-09
41	hMpxV/Brazil/SP-IAL-31/2022	EPI_ISL_14571439	2022-08-05
42	hMpxV/Brazil/SP-IAL-34/2022	EPI_ISL_14571444	2022-08-09
43	hMpxV/Brazil/SP-IAL-35/2022	EPI_ISL_14622055	2022-08-03
44	hMpxV/Brazil/SP-IAL-36/2022	EPI_ISL_14622520	2022-08-02
45	hMpxV/Brazil/SP-IAL-37/2022	EPI_ISL_14622705	2022-08-04
46	hMpxV/Brazil/SP-IAL-38/2022	EPI_ISL_14622706	2022-08-04
47	hMpxV/Brazil/SP-IAL-39/2022	EPI_ISL_14622707	2022-08-04
48	hMpxV/Brazil/RS-IAL-41/2022	EPI_ISL_14622953	2022-07-29
49	hMpxV/Brazil/SP-IAL-42/2022	EPI_ISL_14622960	2022-07-31
50	hMpxV/Brazil/SP-IAL-43/2022	EPI_ISL_14623175	2022-08-03
51	hMpxV/Brazil/SP-IAL-44/2022	EPI_ISL_14623523	2022-08-03
52	hMpxV/Brazil/SP-IAL-45/2022	EPI_ISL_14623704	2022-08-04
53	hMpxV/Brazil/SP-IAL-46/2022	EPI_ISL_14624411	2022-08-03
54	hMpxV/Brazil/SP-IAL-47/2022	EPI_ISL_14624610	2022-08-03
55	hMpxV/Brazil/SP-IAL-48/2022	EPI_ISL_14624698	2022-08-03
56	hMpxV/Brazil/SP-IAL-49/2022	EPI_ISL_14624832	2022-08-04
57	hMpxV/Brazil/SP-IAL-51/2022	EPI_ISL_14625156	2022-08-04
58	hMpxV/Brazil/SP-IAL-52/2022	EPI_ISL_14625157	2022-08-08
59	hMpxV/Brazil/SP-IAL-53/2022	EPI_ISL_14625190	2022-08-05
60	hMpxV/Brazil/PR-IAL-54/2022	EPI_ISL_14625230	2022-08-03
61	hMpxV/Brazil/PR-IAL-55/2022	EPI_ISL_14625256	2022-08-05
62	hMpxV/Brazil/SP-IAL-56/2022	EPI_ISL_14625282	2022-08-08
63	hMpxV/Brazil/SP-IAL-59/2022	EPI_ISL_14772912	2022-08-18
64	hMpxV/Brazil/SP-IAL-60/2022	EPI_ISL_14772913	2022-08-22
65	hMpxV/Brazil/SP-IAL-61/2022	EPI_ISL_14772914	2022-08-17
66	hMpxV/Brazil/SP-IAL-62/2022	EPI_ISL_14773001	2022-08-22
67	hMpxV/Brazil/SP-IAL-63/2022	EPI_ISL_14809096	2022-08-19
68	hMpxV/Brazil/SP-IAL-77/2022	EPI_ISL_14995580	2022-08-30
69	hMpxV/Brazil/SP-IAL-87/2022	EPI_ISL_14995591	2022-09-01
70	hMpxV/Brazil/SP-IAL-90/2022	EPI_ISL_14995611	2022-09-01
71	hMpxV/Brazil/BA-IAL-92/2022	EPI_ISL_14995619	2022-08-31
72	hMpxV/Brazil/SP-IAL-93/2022	EPI_ISL_14995622	2022-09-01
73	hMpxV/Brazil/SP-IAL-97/2022	EPI_ISL_14995653	2022-09-01
74	hMpxV/Brazil/SP-IAL-98/2022	EPI_ISL_14995723	2022-09-04
75	hMpxV/Brazil/RS-CEVS-CDCT131/2022	EPI_ISL_15165603	2022-08-10
76	hMpxV/Brazil/RS-CEVS-CDCT148/2022	EPI_ISL_15165604	2022-08-10
77	hMpxV/Brazil/RS-CEVS-CDCT184/2022	EPI_ISL_15165608	2022-08-11
78	hMpxV/Brazil/RS-CEVS-CDCT45/2022	EPI_ISL_15165614	2022-08-01
79	hMpxV/Brazil/RS-CEVS-CDCT71/2022	EPI_ISL_15165618	2022-08-04
80	hMpxV/Canada/AB-APL-01/2022	EPI_ISL_13194516	2022-05-31
81	hMpxV/Canada/AB-APL-02/2022	EPI_ISL_13269478	2022-06-06
82	hMpxV/Canada/un-NML-2837/2022	EPI_ISL_13408809	2022-05-18
83	hMpxV/Canada/un-NML-2861/2022	EPI_ISL_13408827	2022-05-18
84	hMpxV/Canada/un-NML-2897/2022	EPI_ISL_13408849	2022-05-20
85	hMpxV/Canada/un-NML-3467/2022	EPI_ISL_13544233	2022-06-04
86	hMpxV/Canada//un-NML-3530/2022	EPI_ISL_13827275	2022-06-03
87	hMpxV/Canada/un-NML-3657/2022	EPI_ISL_13908340	2022-06-10
88	hMpxV/Canada/un-NML-3817/2022	EPI_ISL_14050451	2022-06-16
89	hMpxV/Canada/un-NML-3972/2022	EPI_ISL_14587544	2022-07-04
90	hMpxV/Canada/un-NML-3975/2022	EPI_ISL_14587545	2022-07-04
91	hMpxV/Canada/un-NML-4017/2022	EPI_ISL_14587548	2022-07-05
92	hMpxV/Canada/un-NML-4103/2022	EPI_ISL_14594043	2022-07-04

Sample no.	Virus name	Accession no.	Collection date
93	hMpxV/Canada/un-NML-4140/2022	EPI_ISL_14594051	2022-06-30
94	hMpxV/Canada/un-NML-4150/2022	EPI_ISL_14594054	2022-07-05
95	hMpxV/Chile/RM-ISP-75625/2022	EPI_ISL_14224334	2022-06-16
96	hMpxV/DRC/USAMRIID-06-0950/2006	EPI_ISL_13056233	2006-09-10
97	hMpxV/DRC/USAMRIID-06-1076/2006	EPI_ISL_13056238	2006-11-30
98	hMpxV/DRC/USAMRIID-07-0045/2006	EPI_ISL_13056239	2006-12-14
99	hMpxV/DRC/USAMRIID-07-0092/2006	EPI_ISL_13056241	2006-12-26
100	hMpxV/DRC/USAMRIID-07-0093/2006	EPI_ISL_13056242	2006-12-26
101	hMpxV/DRC/USAMRIID-07-0283/2007	EPI_ISL_13056246	2007-02-26
102	hMpxV/DRC/USAMRIID-07-0287/2007	EPI_ISL_13056248	2007-03-20
103	hMpxV/DRC/USAMRIID-07-0354/2007	EPI_ISL_13056251	2007-04-20
104	hMpxV/DRC/USAMRIID-07-0514/2007	EPI_ISL_13056254	2007-06-30
105	hMpxV/DRC/USAMRIID-07-0662/2007	EPI_ISL_13056255	2007-09-04
106	hMpxV/France/un-VGEMI-HCL0001/2022	EPI_ISL_13052287	2022-05-22
107	hMpxV/France/un-IHU-00001/2022	EPI_ISL_14863048	2022-06-13
108	hMpxV/France/un-IHU-00002/2022	EPI_ISL_14863050	2022-07-01
109	hMpxV/France/un-IHU-00003/2022	EPI_ISL_14863051	2022-07-04
110	hMpxV/France/un-IHU-00004/2022	EPI_ISL_14863052	2022-07-04
111	hMpxV/France/un-IHU-00005/2022	EPI_ISL_14863053	2022-07-04
112	hMpxV/France/un-IHU-00007/2022	EPI_ISL_14863055	2022-07-06
113	hMpxV/France/un-IHU-00009/2022	EPI_ISL_14863057	2022-07-07
114	hMpxV/France/un-IHU-00011/2022	EPI_ISL_14863059	2022-07-19
115	hMpxV/Germany/BE-ChVir28656/2022	EPI_ISL_13889435	2022-06-21
116	hMpxV/Germany/BE-ChVir28154/2022	EPI_ISL_13889436	2022-05-28
117	hMpxV/Germany/BE-ChVir28679/2022	EPI_ISL_13889438	2022-06-22
118	hMpxV/Germany/BE-ChVir28689/2022	EPI_ISL_13889439	2022-06-24
119	hMpxV/Germany/BE-ChVir28707/2022	EPI_ISL_13889440	2022-06-25
120	hMpxV/Germany/BE-ChVir28474/2022	EPI_ISL_13889441	2022-06-12
121	hMpxV/Germany/BE-ChVir28149/2022	EPI_ISL_13889446	2022-05-28
122	hMpxV/Germany/BE-ChVir28152/2022	EPI_ISL_13889660	2022-05-29
123	hMpxV/Germany/BE-ChVir28292/2022	EPI_ISL_13890135	2022-05-30
124	hMpxV/Germany/BE-ChVir28138/2022	EPI_ISL_13890273	2022-05-20
125	hMpxV/Italy/FVG-AreaSP-01/2022	EPI_ISL_13052295	2022-05-25
126	hMpxV/Italy/un-INMI-Pt2/2022	EPI_ISL_13251120	2022-05-19
127	hMpxV/Italy/un-INMI-Pt4/2022	EPI_ISL_13331713	2022-05-21
128	hMpxV/Italy/APU-IZSPB-0001/2022	EPI_ISL_13362760	2022-06-08
129	hMpxV/Italy/PIE-OAS-02530345/2022	EPI_ISL_13502582	2022-06-13
130	hMpxV/Italy/SIC-AOUP-UNIPA_VL/2022	EPI_ISL_14783237	2022-08-09
131	hMpxV/Italy/SIC-AOUP-UNIPA_DSPM/2022	EPI_ISL_15055820	2022-08-09
132	hMpxV/Mexico/NLE-UANL-001/2022	EPI_ISL_13607904	2022-06-28
133	hMpxV/Mexico/CMX-InDRE-IBT-001/2022	EPI_ISL_13624509	2022-05-27
134	hMpxV/Netherlands/un-EMC-NL008/2022	EPI_ISL_13822667	2022-06-27
135	hMpxV/Netherlands/un-EMC-NL010/2022	EPI_ISL_13822669	2022-06-27
136	hMpxV/Netherlands/un-EMC-NL011/2022	EPI_ISL_13822718	2022-06-24
137	hMpxV/Netherlands/un-EMC-NL017/2022	EPI_ISL_14254435	2022-07-29
138	hMpxV/Netherlands/un-EMC-NL018/2022	EPI_ISL_14254436	2022-07-22
139	hMpxV/Netherlands/un-EMC-NL019/2022	EPI_ISL_14254437	2022-07-25
140	hMpxV/Netherlands/NH-AUMC-0060/2022	EPI_ISL_14752293	2022-08-05
141	hMpxV/Netherlands/un-EMC-NL026/2022	EPI_ISL_14810370	2022-08-04
142	hMpxV/Netherlands/un-EMC-NL029/2022	EPI_ISL_14810406	2022-08-03
143	hMpxV/Nigeria/CDC-01/2017	EPI_ISL_13056275	2017-11-09
144	hMpxV/Nigeria/CDC-02/2017	EPI_ISL_13056276	2017-11-09
145	hMpxV/Nigeria/CDC-03/2017	EPI_ISL_13056277	2017-12-06
146	hMpxV/Nigeria/CDC-04/2017	EPI_ISL_13056278	2017-11-30
147	hMpxV/Nigeria/CDC-05/2017	EPI_ISL_13056279	2017-11-09
148	hMpxV/Nigeria/CDC-06/2017	EPI_ISL_13056280	2017-11-01
149	hMpxV/Peru/LIM-INS-002/2022	EPI_ISL_13651348	2022-06-27
150	hMpxV/Peru/LIM-INS-003/2022	EPI_ISL_13651349	2022-06-27
151	hMpxV/Peru/TAC-INS-023/2022	EPI_ISL_14207738	2022-07-12
152	hMpxV/Peru/LAL-INS-060/2022	EPI_ISL_14445101	2022-07-22
153	hMpxV/Peru/LOR-INS-039/2022	EPI_ISL_14445152	2022-07-12
154	hMpxV/Peru/PIU-INS-051/2022	EPI_ISL_14445153	2022-07-12
155	hMpxV/Peru/LOR-INS-087/2022	EPI_ISL_14584274	2022-08-01
156	hMpxV/Peru/CUS-INS-111/2022	EPI_ISL_14584275	2022-08-03
157	hMpxV/Peru/LIM-INS-088/2022	EPI_ISL_14584282	2022-07-26
158	hMpxV/Peru/CAL-INS-116/2022	EPI_ISL_14584292	2022-07-25
159	hMpxV/Peru/HUC-INS-121/2022	EPI_ISL_14818783	2022-08-09
160	hMpxV/Peru/LIM-INS-131/2022	EPI_ISL_14818793	2022-08-12
161	hMpxV/Peru/ARE-INS-139/2022	EPI_ISL_14818801	2022-08-10

Sample no.	Virus name	Accession no.	Collection date
162	hMpxV/Peru/UCA-INS-142/2022	EPI_ISL_14818804	2022-08-08
163	hMpxV/Peru/LAL-INS-145/2022	EPI_ISL_14818807	2022-08-12
164	hMpxV/Portugal/INSA-PT0007/2022	EPI_ISL_13052269	2022-05-15
165	hMpxV/Portugal/INSA-PT0023/2022	EPI_ISL_13056892	2022-05-19
166	hMpxV/Portugal/INSA-PT0028/2022	EPI_ISL_13056893	2022-05-18
167	hMpxV/Portugal/INSA-PT0040/2022	EPI_ISL_13466461	2022-06-02
168	hMpxV/Portugal/INSA-0081/2022	EPI_ISL_14515201	2022-07-23
169	hMpxV/Portugal/INSA-PT0292/2022	EPI_ISL_14934540	2022-06-09
170	hMpxV/Portugal/INSA-PT0296/2022	EPI_ISL_14934543	2022-06-08
171	hMpxV/Portugal/INSA-PT0539/2022	EPI_ISL_15199672	2022-08-23
172	hMpxV/Portugal/INSA-PT0549/2022	EPI_ISL_15199682	2022-08-26
173	hMpxV/Portugal/INSA-PT0573/2022	EPI_ISL_15199706	2022-09-06
174	hMpxV/Portugal/INSA-PT0383/2022	EPI_ISL_15199739	2022-07-12
175	hMpxV/Portugal/INSA-PT0387/2022	EPI_ISL_15199743	2022-07-12
176	hMpxV/Portugal/INSA-PT0431/2022	EPI_ISL_15199776	2022-08-01
177	hMpxV/Spain/un-ITER-0001b/2022	EPI_ISL_13331717	2022-05-31
178	hMpxV/Spain/AN-HUCSC-00001/2022	EPI_ISL_13339105	2022-06-07
179	hMpxV/Spain/CT-HUVH-60425/2022	EPI_ISL_13363142	2022-06-10
180	hMpxV/Spain/MD-HULP-8887/2022	EPI_ISL_13449965	2022-06-06
181	hMpxV/Spain/MD-HULP-4061/2022	EPI_ISL_13449966	2022-05-20
182	hMpxV/Switzerland/un-UHG-38134631/2022	EPI_ISL_13052274	2022-05-19
183	hMpxV/Switzerland/un-UHG-38156923/2022	EPI_ISL_13052285	2022-05-24
184	hMpxV/Switzerland/ZH-UZH-IMV-3ba6449f/2022	EPI_ISL_13251157	2022-06-01
185	hMpxV/Switzerland/ZH-UZH-IMV-3ba64538/2022	EPI_ISL_13251723	2022-06-02
186	hMpxV/England/UKHSA-5/2022	EPI_ISL_13958697	2022-05-16
187	hMpxV/United_Kingdom/UKHSA-23/2022	EPI_ISL_14439728	2022-05-25
188	hMpxV/United_Kingdom/UKHSA-42/2022	EPI_ISL_14439747	2022-07-07
189	hMpxV/United_Kingdom/UKHSA-54/2022	EPI_ISL_14439759	2022-05-16
190	hMpxV/United_Kingdom/UKHSA-69/2022	EPI_ISL_14439774	2022-06-30
191	hMpxV/United_Kingdom/UKHSA-70/2022	EPI_ISL_14439775	2022-06-29
192	hMpxV/United_Kingdom/UKHSA-71/2022	EPI_ISL_14439776	2022-06-29
193	hMpxV/United_Kingdom/UKHSA-72/2022	EPI_ISL_14439777	2022-06-28
194	hMpxV/United_Kingdom/UKHSA-74/2022	EPI_ISL_14439779	2022-07-06
195	hMpxV/United_Kingdom/UKHSA-77/2022	EPI_ISL_14439782	2022-07-05
196	hMpxV/United_Kingdom/UKHSA-79/2022	EPI_ISL_14439784	2022-05-25
197	hMpxV/United_Kingdom/UKHSA-80/2022	EPI_ISL_14439785	2022-07-06
198	hMpxV/USA/UT-UPHL-82200022/2022	EPI_ISL_13052288	2022-05-20
199	hMpxV/USA/CA-CDPH-000005/2022	EPI_ISL_13993738	2022-06-07
200	hMpxV/USA/NY-URMC-2207A101/2022	EPI_ISL_14003930	2022-07-11
201	hMpxV/USA/NY-URMC-2207A102/2022	EPI_ISL_14251112	2022-07-14
202	hMpxV/USA/NE-EAOH-25/2022	EPI_ISL_14326644	2022-06-25
203	hMpxV/USA/CT-Yale-00005/2022	EPI_ISL_14526942	2022-07-11
204	hMpxV/USA/CT-Yale-00015/2022	EPI_ISL_14526949	2022-07-13
205	hMpxV/USA/CA-CDPH-000007/2022	EPI_ISL_14736400	2022-06-21
206	hMpxV/USA/NE-UNMC-83/2022	EPI_ISL_14752090	2022-07-27
207	hMpxV/USA/OK-UN-21/2022	EPI_ISL_14804638	2022-06-14
208	hMpxV/USA/OK-UN-41/2022	EPI_ISL_14804639	2022-07-13
209	hMpxV/USA/OK-UN-149/2022	EPI_ISL_14804647	2022-08-11
210	hMpxV/USA/CA-LACPHL-MA00050/2022	EPI_ISL_14818585	2022-07-20
211	hMpxV/USA/NE-UNMC-154/2022	EPI_ISL_14977307	2022-08-12
212	hMpxV/USA/NE-UNMC-181/2022	EPI_ISL_14977308	2022-08-17
213	hMpxV/USA/NC-UNC-0003/2022	EPI_ISL_15076180	2022-08-04
214	hMpxV/USA/CA-LACPHL-MA00103/2022	EPI_ISL_15120452	2022-08-17
215	hMpxV/USA/CA-LACPHL-MA00146/2022	EPI_ISL_15120496	2022-09-02
216	hMpxV/USA/IL-IDPH-002/2022	EPI_ISL_15158316	2022-08-04
217	hMpxV/USA/IL-IDPH-053/2022	EPI_ISL_15158367	2022-07-04
218	hMpxV/USA/IL-IDPH-073/2022	EPI_ISL_15158387	2022-06-30

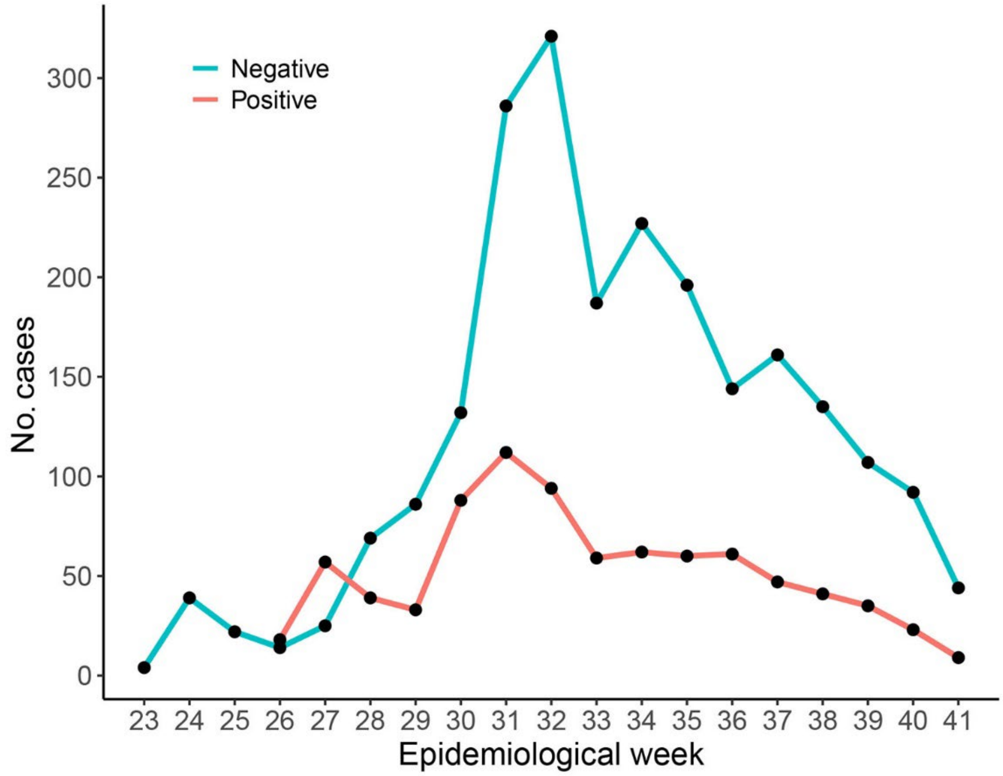

Appendix 1 Figure 1. Total number of negative and positive mpox cases per epidemiologic week in Minas Gerais, Brazil, 2022.

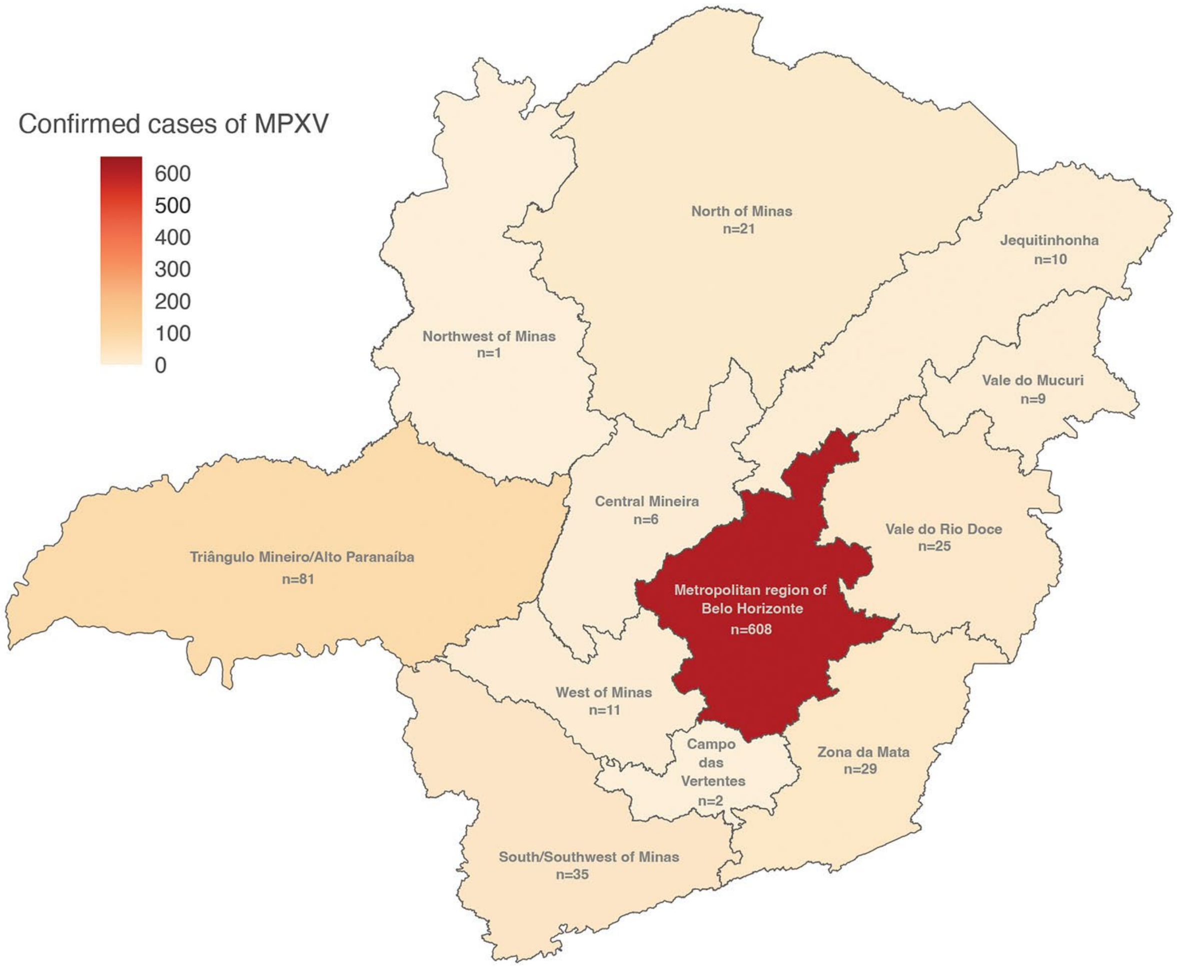

Appendix 1 Figure 2. Map of the state of Minas Gerais showing the number of confirmed mpox cases per mesoregion from epidemiologic week 23 (June 5–11) to 41 (October 9–15), 2022.

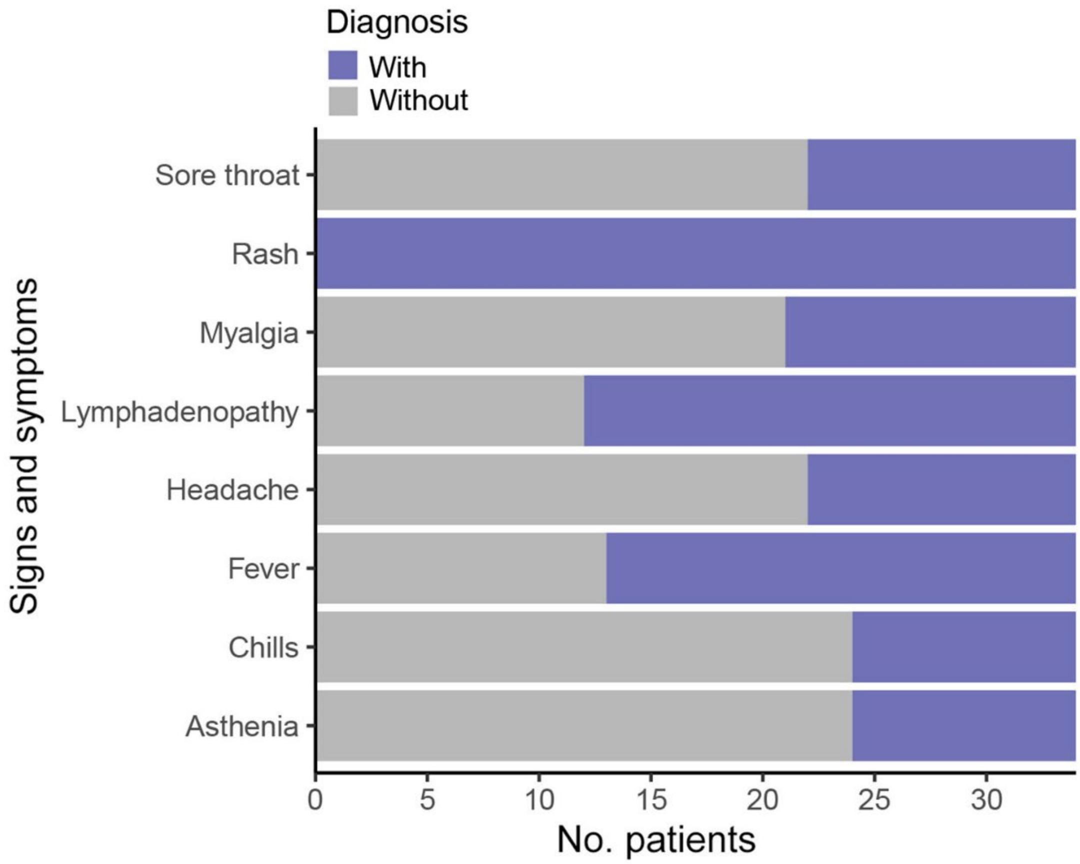

Appendix 1 Figure 3. Signs and symptoms reported in the clinical records of patients with sequenced samples used to obtain monkeypox virus genome, Minas Gerais, Brazil.
